# Virtual reality and motor imagery for early post-stroke rehabilitation

**DOI:** 10.1186/s12938-023-01124-9

**Published:** 2023-07-05

**Authors:** Chi S. Choy, Qiang Fang, Katrina Neville, Bingrui Ding, Akshay Kumar, Seedahmed S. Mahmoud, Xudong Gu, Jianming Fu, Beth Jelfs

**Affiliations:** 1 Department of Biomedical Engineering, Shantou University, Shantou, China; 2 Department of Electrical, Electronic & Systems Engineering, University of Birmingham, Birmingham, UK; 3 School of Engineering, RMIT University, Melbourne, Australia; 4 Rehabilitation Center, Jiaxing 2nd Hospital, Jiaxing, 314000 China

**Keywords:** Stroke, Motor imagery, Virtual reality, EEG, Rehabilitation, Motor recovery, Neuroplasticity, Brunnstrom, Spectral analysis, Entropy

## Abstract

**Background:**

Motor impairment is a common consequence of stroke causing difficulty in independent movement. The first month of post-stroke rehabilitation is the most effective period for recovery. Movement imagination, known as motor imagery, in combination with virtual reality may provide a way for stroke patients with severe motor disabilities to begin rehabilitation.

**Methods:**

The aim of this study is to verify whether motor imagery and virtual reality help to activate stroke patients’ motor cortex. 16 acute/subacute (< 6 months) stroke patients participated in this study. All participants performed motor imagery of basketball shooting which involved the following tasks: listening to audio instruction only, watching a basketball shooting animation in 3D with audio, and also performing motor imagery afterwards. Electroencephalogram (EEG) was recorded for analysis of motor-related features of the brain such as power spectral analysis in the $\alpha$ and $\beta$ frequency bands and spectral entropy. 18 EEG channels over the motor cortex were used for all stroke patients.

**Results:**

All results are normalised relative to all tasks for each participant. The power spectral densities peak near the $\alpha$ band for all participants and also the $\beta$ band for some participants. Tasks with instructions during motor imagery generally show greater power spectral peaks. The *p*-values of the Wilcoxon signed-rank test for band power comparison from the 18 EEG channels between different pairs of tasks show a 0.01 significance of rejecting the band powers being the same for most tasks done by stroke subjects. The motor cortex of most stroke patients is more active when virtual reality is involved during motor imagery as indicated by their respective scalp maps of band power and spectral entropy.

**Conclusion:**

The resulting activation of stroke patient’s motor cortices in this study reveals evidence that it is induced by imagination of movement and virtual reality supports motor imagery. The framework of the current study also provides an efficient way to investigate motor imagery and virtual reality during post-stroke rehabilitation.

**Supplementary Information:**

The online version contains supplementary material available at 10.1186/s12938-023-01124-9.

## Background

Stroke is a brain lesion which generally causes disability and even death [1, 2]. Motor impairment is a common consequence of stroke affecting stroke patients’ ability to live independently [3, 4]. The major mechanism behind post-stroke recovery is neuroplasticity which rewires the neural network of the brain [5, 6]. Early motor rehabilitation is essential to effectively restore motor function of stroke patients because neuroplasticity is most active within the first month post-stroke [2, 6, 7]. Initially, mobility of stroke patients is minimal; often they are not able to instigate any movement [2, 8]. There are six Brunnstrom motor recovery stages (BMRS) which describe different levels of mobility[2]. Stroke patients who cannot initiate any movement from affected body parts are classified in stage 1 of the BMRS [2]. Conventional post-stroke rehabilitation that relies on physical movement may be ineffective during the early stage of post-stroke motor recovery because stroke patients are often severely paralysed and unable to participate in physical rehabilitation [2, 8, 9].

Motor imagery (MI) is the mental representation of a body movement [6]. In MI, a patient is required to mentally rehearse a movement without its physical execution [3, 4]. It was suggested that MI could promote recovery of the lesioned brain areas using functional and other neuronal networks; hence, MI appears to be an effective alternative therapy for early post-stroke motor rehabilitation [5, 7]. However, MI requires training and may be challenging particularly for stroke patients [10].

It was shown that observing an action may activate the motor cortex and promote motor learning; thus, facilitating neural recovery [3, 11]. This is due to the mirror neurons being activated during both action execution and observation [3, 11]. The mirror neuron system assists the observer to imitate an observed action; hence, there may be an overlap between action observation (AO) and the process of performing a physical movement [3, 11]. It was also reported that AO via virtual reality (VR) technology could assist stroke patients to focus on MI tasks by visually simulating real movements within an immersive environment, minimising distractions from the surroundings, thus, potentially reducing the difficulty of conventional MI [10, 12]. VR technology has also been shown to assist stroke patients in a minimally conscious state to perform MI [9]. As a result, combining MI and AO for performing the same movement may enhance activation of the motor cortex and facilitate motor recovery of stroke patients, especially in stage 1 of the BMRS [4, 5, 10]. Despite there being positive evidence of VR-assisted MI in post-stroke rehabilitation, the experimental protocol of different studies is not standardised and involves various VR machineries [8, 10, 12]. The findings of different VR-MI studies are not conclusive though promising.

Physiological measure of MI recorded by electroencephalogram (EEG) provides a relatively accessible and objective way to measure brain signals induced by MI with a high temporal resolution [10, 12–16]. In this study, we apply filters as well as both EEG spectra and entropy analyses to investigate whether MI and VR may help to activate the brain areas responsible for motor functions; thus, potentially promoting motor recovery.

## Results

**Fig. 1:**
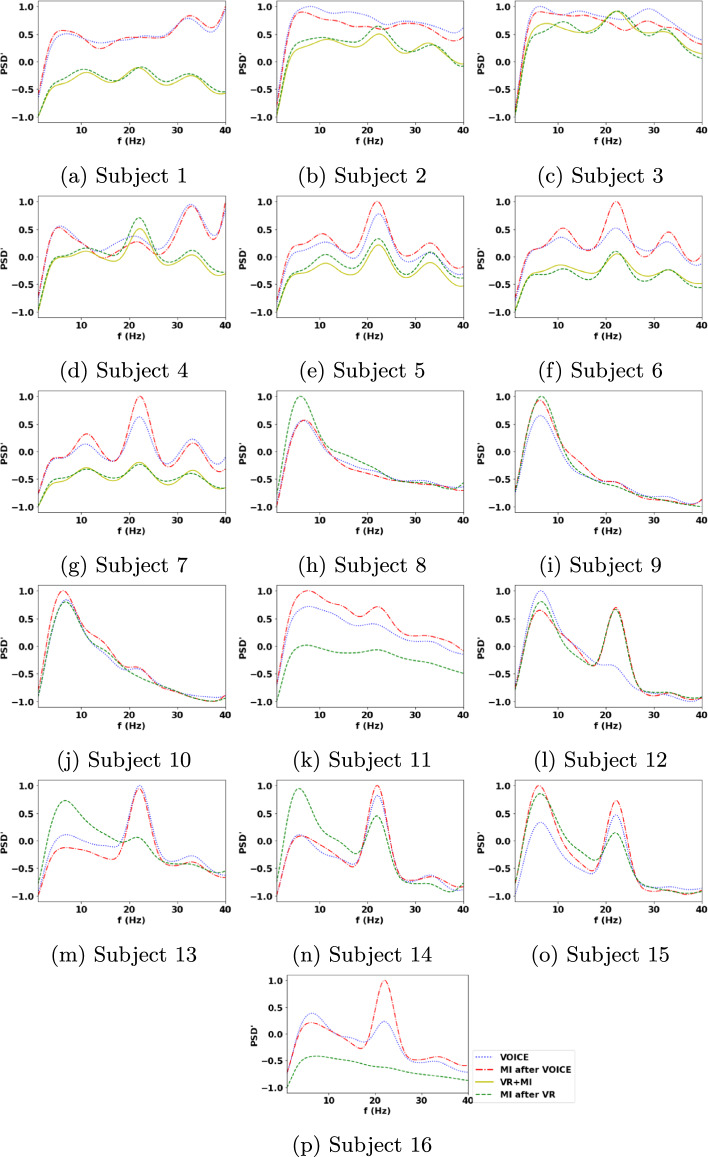
Periodograms for each of the subjects from Experiment 1 (**a**–**g**) and Experiment 2 (**h**–**p**) showing their normalised power spectral densities ($PSD'$) across frequencies 1 to 40 Hz. The legend for all plots is shown at the bottom right of this figure

**Fig. 2:**
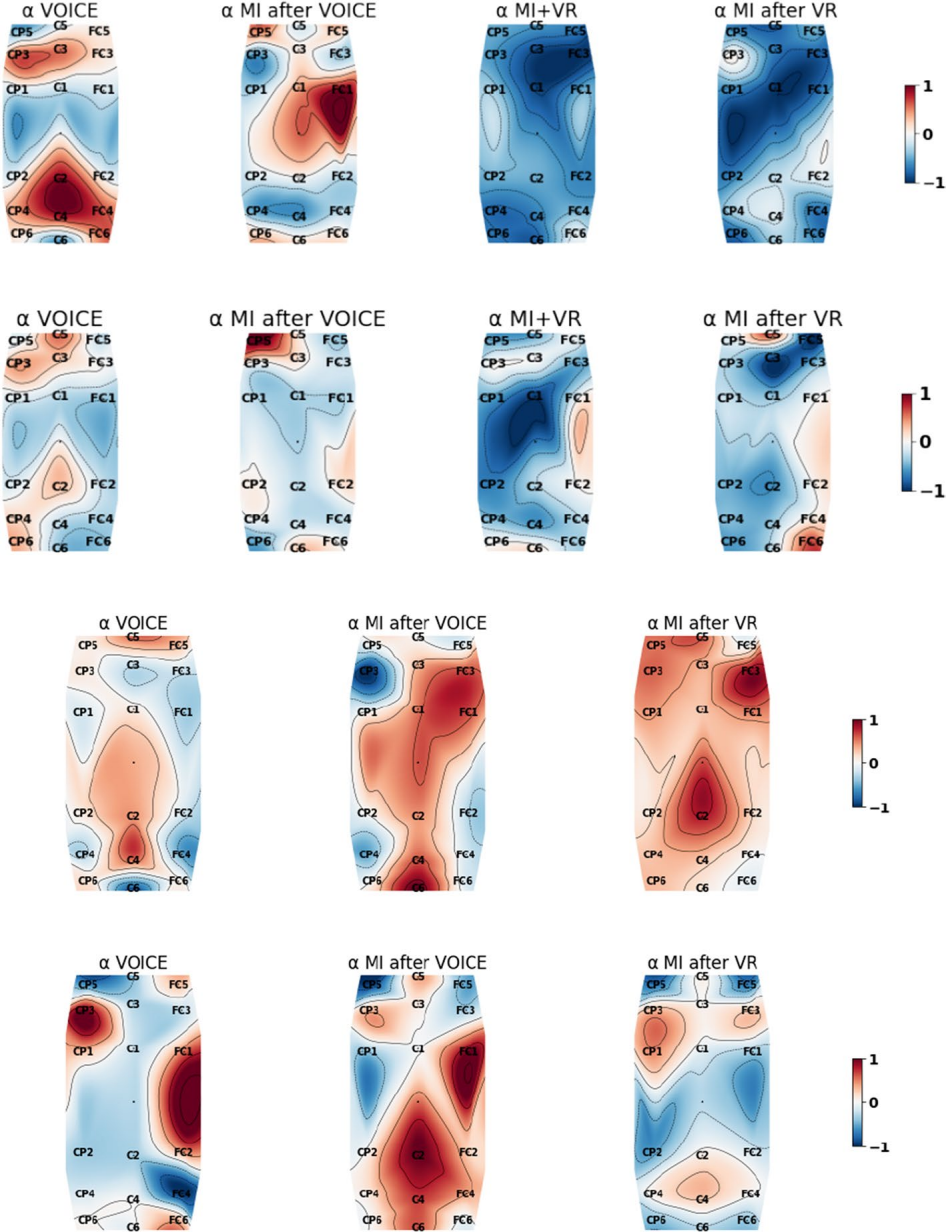
$\alpha$ band power scalp maps of stroke patients 2, 3, 8 and 10 (top row to bottom row) showing the intensity variations normalised relative to all classes from Experiments 1 and 2, respectively. Red is 1 = maximum; blue is −1 = minimum

**Fig. 3:**
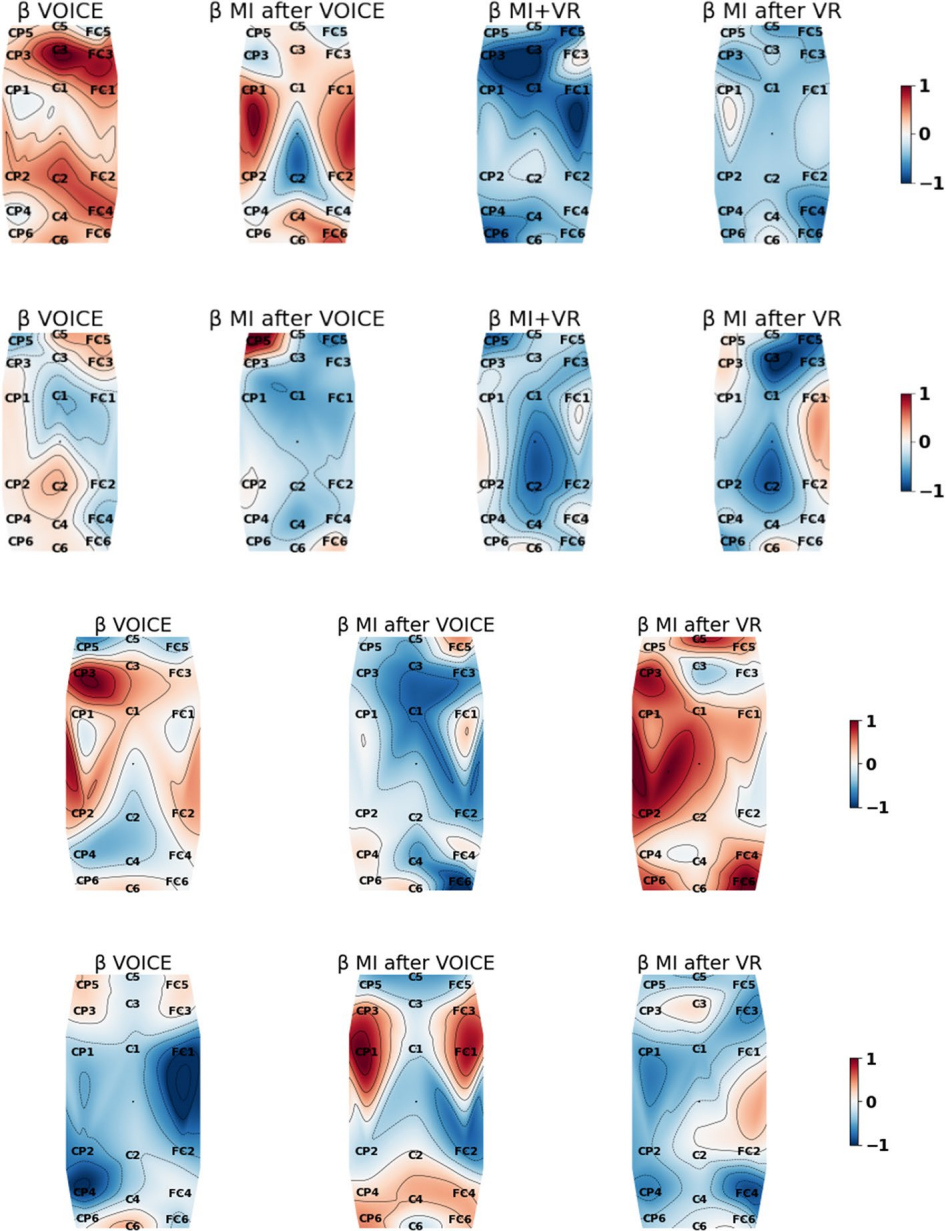
$\beta$ band power scalp maps of stroke patients 2, 3, 8 and 10 (top row to bottom row) showing the intensity variations normalised relative to all classes from Experiments 1 and 2, respectively. Red is 1 = maximum; blue is −1 = minimum

**Fig. 4:**
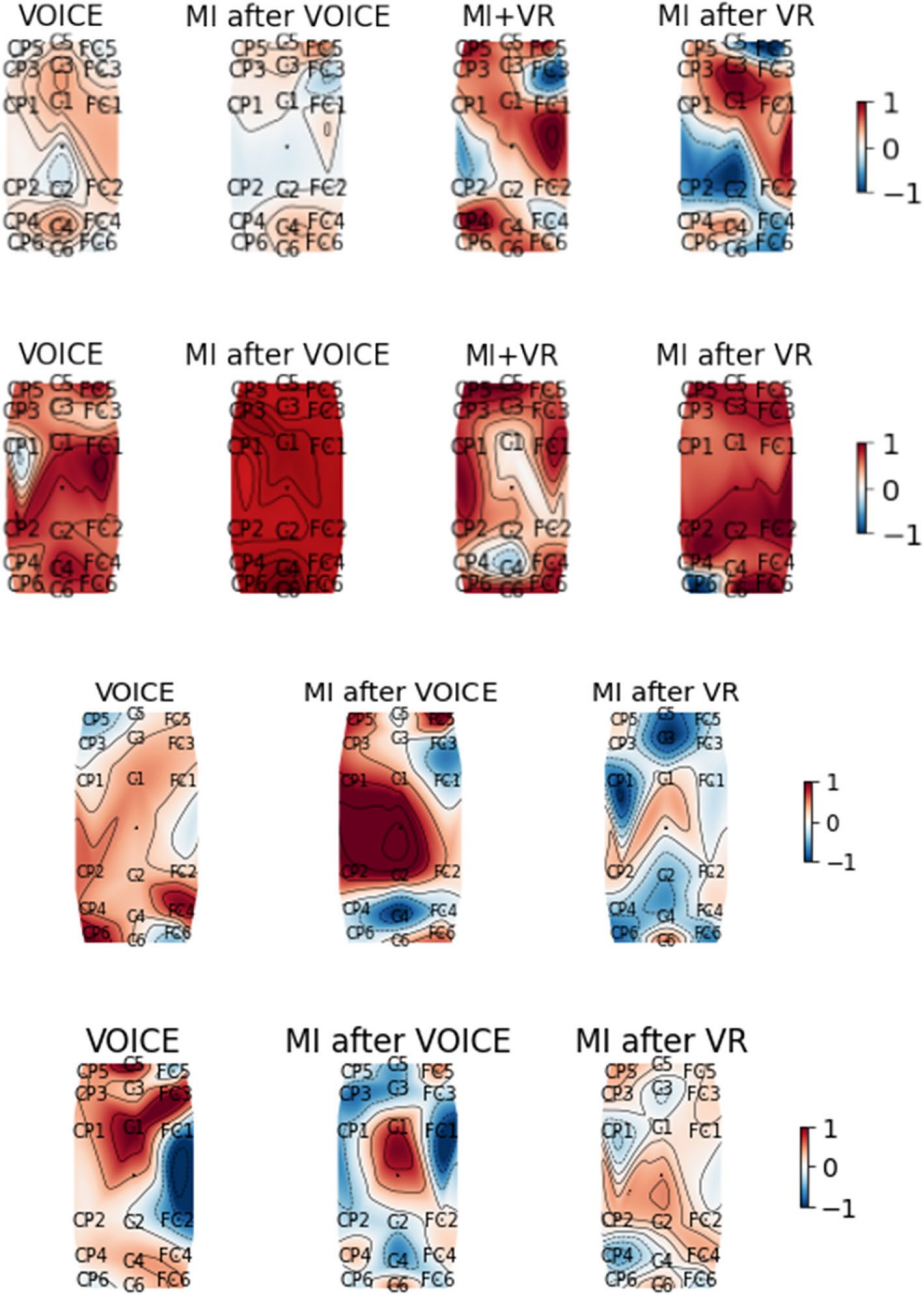
Spectral entropy scalp maps of stroke patients 2, 3, 8 and 10 (top row to bottom row) for frequencies 1–40 Hz showing the intensity variations normalised relative to classes from Experiments 1 and 2, respectively. Red is 1 = maximum; blue is – 1 = minimum

**Fig. 5:**
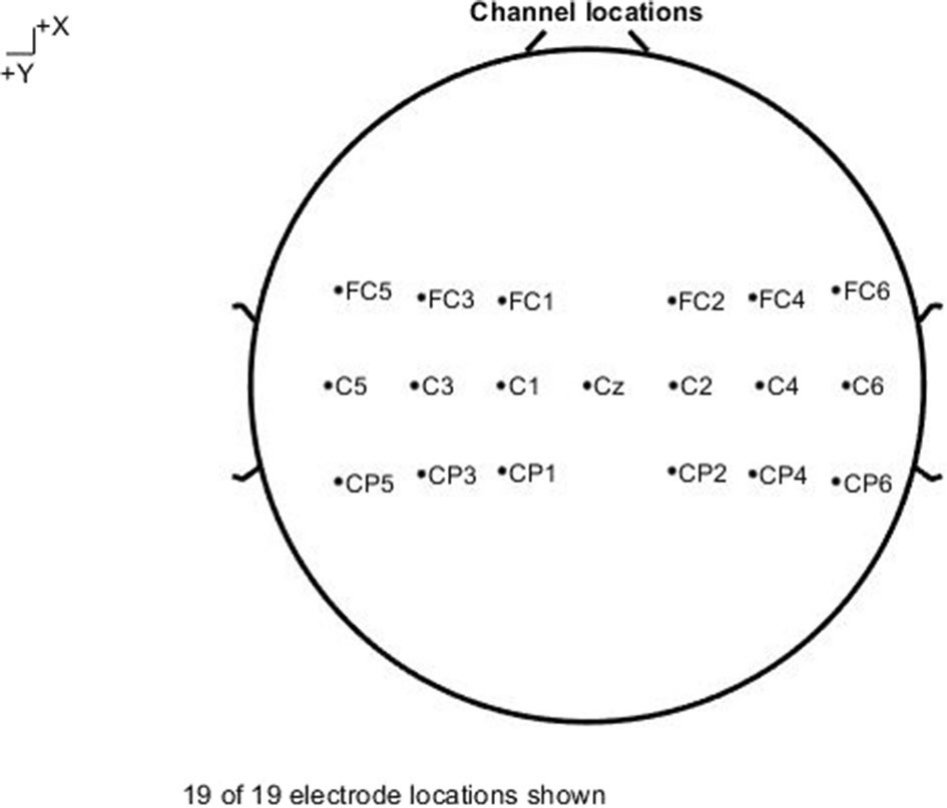
Diagram showing the electrode position distribution for stroke subjects

The 18 EEG channels covering the motor brain areas shown in Fig. 5 are considered in computing the periodograms and band powers for stroke patients as movement processes mainly involve the motor cortex [8, 10]. Figure 1 shows all subjects’ epoch-averaged periodograms normalised with respect to the subjects’ own experimental tasks in this study: VOICE, MI after VOICE, VR+MI, and MI after VR, with 1 = the maximum and −1 = the minimum. Table 1 presents the p-values of the Wilcoxon signed-rank test for comparing the $\alpha$ and $\beta$ band powers associated with different pairs of tasks performed by the stroke patients. Figures 2 and 3 are, respectively, the normalised $\alpha$ and $\beta$ band power scalp maps relative to all classes in Experiment 1 for stroke patients 2 and 3 and in Experiment 2 for stroke patients 8 and 10. Figure 4 shows the spectral entropy scalp maps normalised to all classes in Experiments 1 and 2, respectively.

**Table 1:** Results of the Wilcoxon signed-ranked test between different tasks in the $\alpha$ and $\beta$ frequency bands from the 18 EEG channels on the motor brain areas of stroke subjects

No.	VOICE	VOICE	VOICE	MI after VOICE	MI after VOICE	VR+MI
	MI after VOICE	VR+MI	MI after VR	VR+MI	MI after VR	MI after VR
$\alpha$; Subject						
1	0.899	<**0.001**	<**0.001**	<**0.001**	<**0.001**	0.154
2	0.196	<**0.001**	<**0.001**	<**0.001**	**0.002**	0.442
3	0.640	<**0.001**	0.130	0.024	0.099	0.551
4	1	0.034	0.039	0.024	0.060	0.417
5	0.014	<**0.001**	**0.001**	<**0.001**	<**0.001**	**0.009**
6	0.054	<**0.001**	**0.001**	<**0.001**	<**0.001**	0.030
7	0.024	<**0.001**	**0.001**	<**0.001**	<**0.001**	0.671
8	0.671	–	**0.001**	–	0.048	–
9	**0.002**	–	<**0.001**	–	0.369	–
10	0.229	–	0.671	–	0.021	–
11	0.012	–	<**0.001**	–	<**0.001**	–
12	0.081	–	**0.006**	–	0.551	–
13	**0.001**	–	<**0.001**	–	<**0.001**	–
14	0.081	–	<**0.001**	–	<**0.001**	–
15	<**0.001**	–	<**0.001**	–	0.043	–
16	0.108	–	<**0.001**	–	<**0.001**	–
$\beta$; Subject						
1	0.734	<**0.001**	<**0.001**	<**0.001**	<**0.001**	0.265
2	0.016	<**0.001**	<**0.001**	<**0.001**	<**0.001**	0.054
3	0.018	**0.002**	0.060	0.932	0.495	0.865
4	0.016	<**0.004**	0.393	0.468	0.119	0.016
5	0.021	<**0.001**	<**0.001**	**0.001**	**0.001**	**0.001**
6	0.039	**0.001**	<**0.001**	<**0.001**	<**0.001**	0.167
7	0.027	<**0.001**	<**0.001**	<**0.001**	<**0.001**	0.766
8	0.054	–	0.014	–	<**0.001**	–
9	0.030	–	0.090	–	**0.001**	–
10	0.060	–	0.417	–	0.012	–
11	**0.001**	–	<**0.001**	–	<**0.001**	–
12	<**0.001**	–	<**0.001**	–	0.304	–
13	<**0.001**	–	<**0.001**	–	**0.001**	–
14	0.265	–	0.229	–	0.018	–
15	<**0.001**	–	0.212	–	0.012	–
16	<**0.001**	–	<**0.001**	–	<**0.001**	–

Statistically significant results with a *p-value* $<0.01$ are indicated in bold

## Discussion

The experiments of this study aim to guide participants to mentally perform a basketball shooting movement which they cannot perform physically. Bimanual basketball shooting is selected as the MI task to mimic a sport activity involving the upper limbs. Mentally performing a sport involving both hands may actively promote both hemispheres of the brain to be activated, maximising brain activity of the motor cortex, especially for non-experts [3, 6, 17–21]. An upper limb MI is chosen because a larger area of the motor cortex is activated to control upper limbs, thus has been shown to be more effective in motor function recovery than that of lower limbs [3, 6, 22]. The basketball shooting instruction provided in the current study prompts participants to activate their motor cortex by gradually guiding them to imagine the movement in a few steps. Stroke patients have suffered brain damage, so movement instruction should be relatively straightforward and simulate physical movement as much as possible to make the MI task practical [6, 9, 23–27]. A sport exercise shown via video is used to induce a sense of embodiment and self-esteem from the stroke patients by attempting to trigger the neural pathways for motor processes through the patients’ imagination of performing a physical task that appears to be impossible [6, 8, 17, 18, 21, 28–30]. The activation intensity distribution of the brain can be studied by power spectral density (PSD), band power and spectral entropy of the EEG data that have been preprocessed [8, 31–33].

### Power spectral density

Activation of the motor cortex is expected to induce signal peaks predominantly in the alpha (8–12 Hz) and beta (13–30 Hz) frequency bands as they correspond to motor-related processes [10, 33–38]. Periodograms illustrate how each subject’s spectral power distribution changes across different frequencies. A larger magnitude of intensity of spectral power peaks in the periodograms indicates a greater brain activation at the corresponding frequencies. For stroke subjects, there are peaks in the $\alpha$ and $\beta$ frequency bands for all of the tasks. A power peak in either the $\alpha$ or the $\beta$ band detected from the motor cortex can itself be used as an indicator of motor-related processes [10, 33–38]. There is a peak in the 20–25 Hz range within the $\beta$ band in all classes as illustrated in Fig. 1a–p. The spectral power peaks in the $\alpha$ band are greater for MI after VR than those of other classes for subjects 8, 9, 13 and 14, indicating VR assistance for MI. In Experiment 2, there is no assistance provided to subjects performing MI after VR for approximately 5 min; hence, distractions and fatigue may affect some subjects’ MI ability. MI after VOICE shows a distinctively greater peak in the 20–25Hz range than that of both VOICE and MI after VR for subjects 11, 15 and 16, as shown in Fig. 1k, o and p.

VR+MI in Experiment 1 provided visual and audio instructions in 3D while stroke patients perform MI. There is a more prominent peak in 20–25 Hz for VR+MI and MI after VR than that of other tasks for subjects 3 and 4 as shown in Fig. 1c and d. VOICE may only be helpful for subjects 5–7 from Experiment 1 as illustrated by their $PSD'$ peaks in the $\beta$ band. MI after VOICE shows no assistance for half of the subjects in Experiment 1 as indicated by its broad and flat power spectrum as illustrated in Fig. 1a–g. This may again due to attention deficiency when no cues for MI are given at all.

### Band power

Band powers provide an overall representation of PSD patterns. Greater $\alpha$ and $\beta$ band powers correspond to more intense motor-related brain activation[10, 33, 34]. The Wilcoxon signed-rank test is non-parametric which is ideal for comparing two conditions for the same participants without assuming normality of samples[39]. The results indicate that there is a 0.01 significance of rejecting the null hypothesis that VR+MI (or MI after VR) and MI after VOICE have the same band powers for almost all stroke subjects; however, VOICE and MI after VOICE have more similar band powers. Similarly, VR+MI and MI after VR also have more similar band powers as indicated by their p-values not reaching the 0.01 significance level.

Stroke patients 2, 3, 8 and 10 are illustrative of the types of responses seen in all patients. A higher value of band power (red) corresponds to more intense brain activation. The other stroke patients from Experiment 1 show similar brain activity to stroke patients 2 and 3. Most Experiment 1 stroke patients’ brain activities are similar to stroke patient 2’s, with the motor cortex being less active during MI+VR and MI after VR, as shown in the first row of Figs. 2 and 3, and Additional file 1: Figures S1 and S2. This could be attributed to some stroke patients performing visual imagery (VI) because of their lack of understanding of MI, therefore decreasing activity in the motor cortex. Stroke patient 2 may not be able to focus on MI even with VR assistance; whereas, the motor cortex of stroke patient 3 is more active during MI after VR and MI after VOICE in Experiment 1. Performing MI after VR is present appears to help stroke patient 3 to activate the motor cortex near channel C4 as illustrated by more intense $\alpha$ band power.

Stroke patients 8 and 10 share similarities with the other stroke patients from Experiment 2. The motor cortex of stroke patient 8 during MI after VR in Experiment 2 is overall more activated in the $\alpha$ and $\beta$ bands compared to other classes without VR as shown in Figs. 2 and 3. Stroke patient 10 does not reflect any assistance of VR in MI induced brain activation in the motor cortex compared to MI without VR as shown in Figs. 2 and 3.

Stroke patients 3 and 8 are illustrative of most stroke patients’ brain activities in the $\alpha$ and $\beta$ bands, achieving maximum band power for conditions involving VR; whereas, a small number of stroke patients have similar band power scalp maps as stroke patient 10, not activating the motor cortex during MI after VR as illustrated in Additional file 1: Figures S1–S4.

### Spectral entropy

Entropy in a biological process measures the complexity of a physiological signal [40–45]. Brain activation is associated with peaks in the $\alpha$ and $\beta$ band of a power spectrum; hence, spectral entropy is used to study brain activation by analysing power spectral patterns. A power spectrum with a pattern identical to that of a single frequency component such as a sinusoid has the smallest spectral entropy [31, 32]. On the contrary, a flat power spectrum having all frequency component s with equal power like that of white noise corresponds to the greatest spectral entropy [31, 32]. A higher value of spectral entropy represents a more uniform and flatter power spectrum distribution [31, 46, 47]. Spectral entropy is a measure of the regularity of a power spectrum which should be interpreted together with spectral power analysis. A lower value of spectral entropy (blue) is associated with more intense brain activity if the associated band power is closer to maximum.

Most stroke patients’ brain patterns are similar to stroke patients 3 and 8 having the lowest spectral entropy values for VR-assisted MI; whereas, some stroke patients are similar to stroke patient 10 with higher spectral entropy values for VR-assisted MI as shown in Additional file 1: Figures S5 and S6. VR potentially assists most stroke patients to activate their motor cortex during MI, but is ineffective for some stroke patients as indicated by Figs. 2, 3 and 4.

## Conclusions

MI may activate the motor brain areas of stroke patients as deduced by their normalised PSD, band power and spectral entropy computed in this study. The $PSD'$ peaks, maximum band power and minimum spectral entropy are present in the motor-related $\alpha$ and $\beta$ frequency bands for most of the 16 (acute/subacute) stroke patients’ motor cortices during VR-assisted MI indicating more intense brain activation than that of MI alone. The p-values of the Wilcoxon signed-rank test associated with $\alpha$ and $\beta$ band powers between the conditions in Experiments 1 and 2 of this study, respectively, achieve a 0.01 significance for most stroke patients indicating that MI tasks involving VR and without VR do not have the same brain activation pattern. VR is potentially an effective tool for assisting MI performance. MI in combination with VR could be particularly beneficial for stroke patients without other rehabilitative options because of their severe motor impairment. Future research may investigate the effects of fatigue and sensory distractions during shorter or longer MI experiments [26, 27, 48–52]. Nonetheless, the results and framework of this study are useful for future work which may provide new insights in the applicability of MI and VR in stroke rehabilitation.

## Methods

### Experimental protocol

#### Participants

Experiments on stroke patients were conducted at Jiaxing 2nd hospital in China in April and July 2021 [53]. These experiments were approved by the Ethics Committee of Jiaxing 2nd Hospital Rehabilitation Centre in accordance with the Declaration of Helsinki. All stroke patients gave informed consent before participating in the study. The experiment could be terminated whenever the participants felt unwell with symptoms such as nausea. An initial assessment of upper limb mobility of stroke patients based on the BMRS and the mini-mental state examination (MMSE) for cognitive function were performed by medical doctors. All participants’ demographics are shown in Table 2 according to the following enrolment criteria:

i Subjects were over 18 years old.
ii Subjects were in stage I, II or III of the Brunnstrom stages of stroke recovery.
iii Subjects have normal vital signs and with sufficient vision and hearing to follow instructions as determined by medical doctors using the MMSE assessment with a minimum threshold score of 10 [9, 54, 55].

Only adult stroke patients with stable vitals were recruited by clinicians to minimise unforeseen paediatric medical complications. MI is most beneficial for stroke patients with no or minimal physical movement which persists through the first 3 BMRS. Most stroke patients in this study achieve either a MMSE score corresponding to normal cognitive function, i.e. above 25 or even 30, the maximum. Only stroke patients 12 and 15 have a MMSE score in the range 10 to 19 indicating potentially moderate, but not severe, cognitive impairment [9, 54, 55].

**Table 2:** Demographic information for the (Experiments 1 and 2) stroke participants

Subject	Gender	Age	Affected	Stroke	Brunnstrom	Post-stroke	MMSE
No.			Side	Condition	Stage	(months)	Score
Experiment 1							
1	Male	74	Right	Left basal ganglia haemorrhage	I	4	26
2	Male	46	Right	Left basal ganglia haemorrhage	III	5	30
3	Male	52	Right	Left basal ganglia haemorrhage	II	2	30
4	Male	70	Left	Right basal ganglia haemorrhage and parietal ventricular cerebral infarction	II	<1	30
5	Male	71	Left	Right basal ganglia and parietal ventricular foci of encephalomalacia	II	1	30
6	Male	70	Left	Right cerebral peduncle foci infarction	I	1	30
7	Female	63	Right	Left basal ganglia and parietal ventricular multiple scattered foci infarction	III	5	30
Experiment 2							
8	Female	52	Left	Right basal ganglia cerebral haemorrhage	II	4.5	30
9	Male	58	Right	Left basal ganglia ventricular haemorrhage	II	2.5	30
10	Male	48	Left	Brain stem and right corpus callosum cerebral infarction and bilateral paraventricular foci ischaemia	I	1.5	30
11	Female	50	Left	Right basal ganglia haemorrhage	I	2	30
12	Male	68	Right	Bilateral paraventricular and lacunar foci ischaemia	I	2	13
13	Male	61	Right	Bilateral lateral ventricular foci ischaemia	I	1	30
14	Male	37	Right	Left basal ganglia foci infarction	I	1	30
15	Female	86	Right	Left lateral thalamic haemorrhage	II	1	16
16	Female	76	Right	Left basal ganglia lacunar infarction	I	1	30

**Fig. 6:**
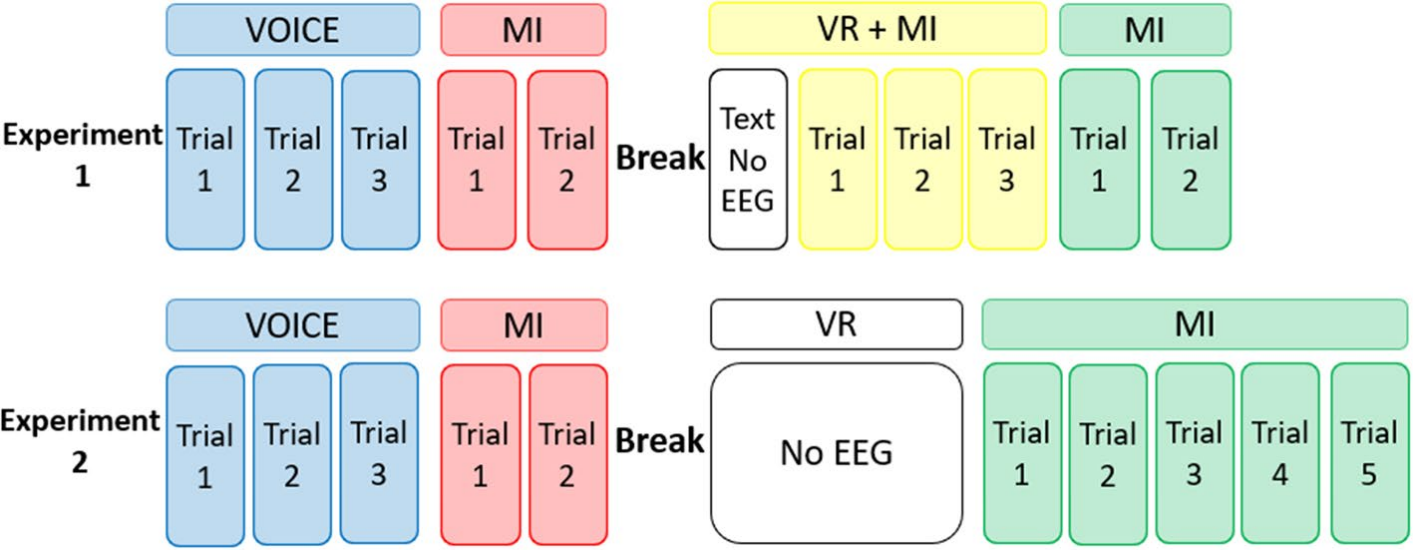
Flowcharts showing the schematic of the experiments outlining the tasks performed by the subjects, with each trial lasting 1 min in duration. There are four tasks in Experiment 1: VOICE, MI after VOICE, VR+MI and MI after VR. There are three tasks in Experiment 2: VOICE, MI after VOICE and MI after VR

**Fig. 7:**
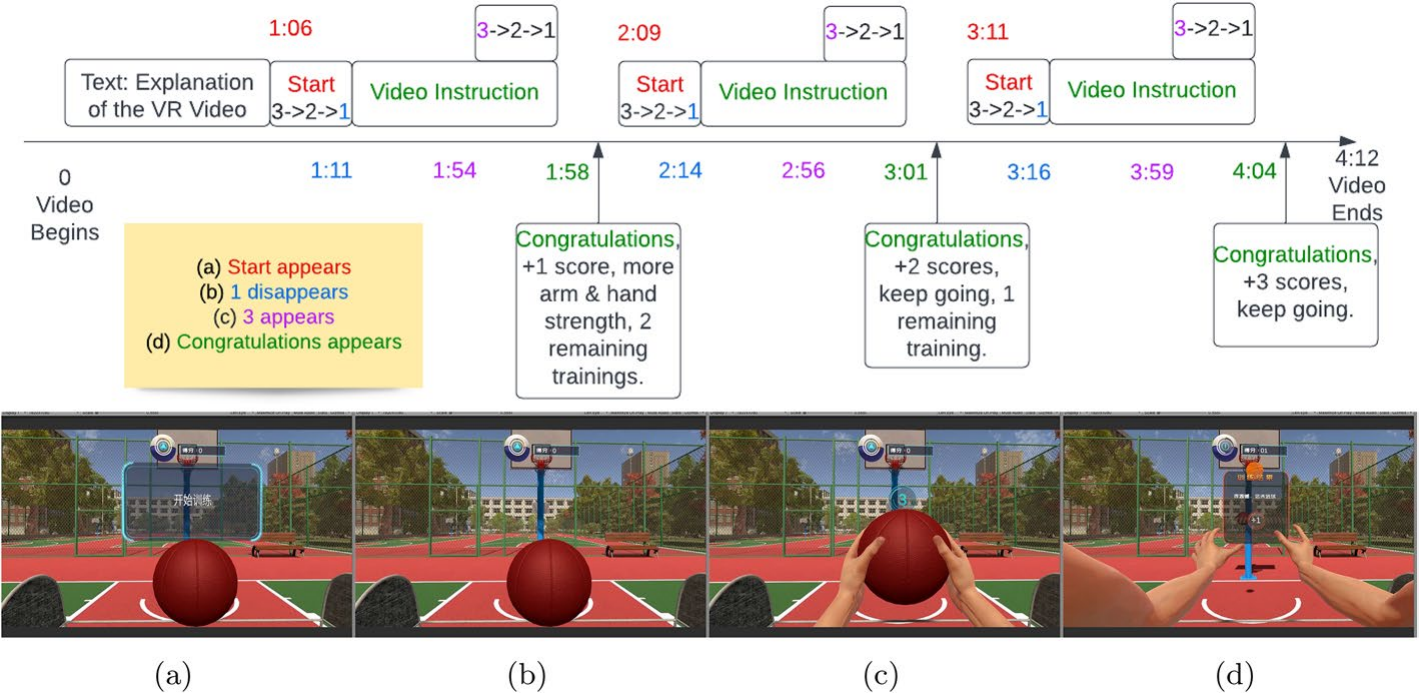
Flowchart showing the timing of the video for the VR+MI task. **a**–**d** are four cues, the colours of the cues are consistent with the corresponding colours of the times at which they occur in the experimental process

#### Data collection

A g.HIamp (from g.tec, Austria) with 80 wet electrode channels arranged in the standard international 10–10 configuration at a sampling rate of 1200 Hz were used to record EEG data from all stroke patients. Figure 5 shows the following 18 EEG channels used for this study covering the motor brain areas: FC1, FC2, FC3, FC4, FC5, FC6, C1, C2, C3, C4, C5, C6, CP1, CP2, CP3, CP4, CP5, and CP6. The reference channel is attached to the left earlobe and Cz is the ground channel.

The EEG collected from each task of the experiment were saved as 1-min files where each file corresponded to 1 trial for stroke patients. The number of trials for stroke patients is shown in Experiments 1 and 2 of Fig. 6.

#### Experimental design

Subjects wore the EEG devices and were lying down on a bed during the experiments. The current study consists of 2 experiments as shown in Fig. 6 illustrating the periods of EEG measurement and their associated experimental tasks. The break period is 1 day for Experiment 1 and 15–20 min for Experiments 2. The (Mandarin Chinese) audio and video instructions, designed by Shantou University using Assembly-CSharp API from Unity[56], were given in a few steps as shown in Fig. 7. For tasks involving VR (in 3D), subjects wore a head-mounted display centred in a VR environment made by HTC VIVE PRO EYE with the helmet-mounted display (HMD) of 1440$\times$1600 resolution per eye and 110$^{\circ }$Field of view. Further details of the respective tasks of the experiments are provided in the following:

**VOICE** The subject wears headphones and listens to the voice instructions (in Chinese) which describes a sequence of movements for the purpose of shooting a basketball with both hands, i.e. hands reaching and holding the ball, lifting the ball, increasing arm strength, then shooting the ball. The voice instruction is played three times, each time lasts for 1 min and it is the same audio used in the video instruction from cues (a) to (d) shown in Fig. 7. Three trials were performed for all experiments.

**MI after VOICE** The subject was asked to imagine the movement associated with the voice instruction immediately after the end of the voice instruction. All experiments had 2 trials performed.

**VR+MI** In addition to the voice guidance, the subject wore the head-mounted display and observed the animation corresponding to the voice instruction for shooting a basketball while imagining the associated movement simultaneously. Figure 7 illustrates the timeline of the video instruction with cues showing the general structure of the whole video. Firstly, there is a text instruction which lasts for 1 min. Secondly, a 3-2-1 countdown appears, then the video is played in sync with the voice instruction having another 3-2-1 countdown before shooting the basketball from cues (a) to (d) of Fig. 7. Finally, a congratulatory message follows the basketball shooting. There were 3 trials for VR+MI as only text instruction was presented in the first minute of EEG recording as shown in Experiment 1 of Fig 6.

**MI after VR** In this task, the subject performs MI of shooting the basketball after watching the corresponding 3D video from the head-mounted display. Each minute of EEG recording is considered 1 trial as depicted in Fig. 6.

### Data preprocessing

**Fig. 8:**
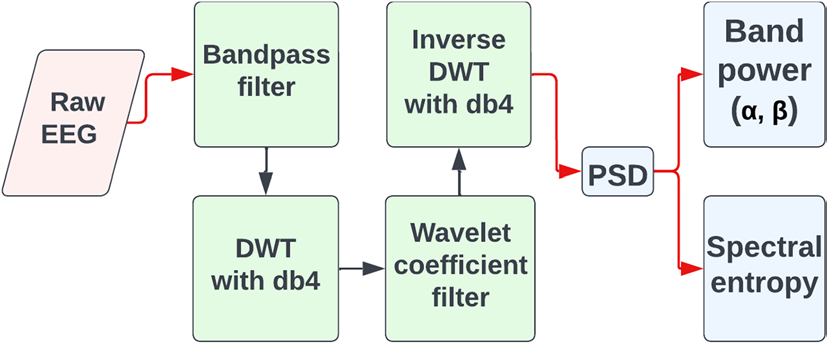
Pipeline for EEG data preprocessing (green) and analysis (blue) applied in this study

Figure 8 shows the general procedure for processing EEG data in the current study. Raw EEG is extracted as input data. At the beginning of each trial, the EEG recordings contain noise interference caused by the machinery or other sources; furthermore, trial recordings do not contain exactly the same number of samples. As a result, only samples from the first $6{th}$ second to the $51{st}$ second of each trial are considered for noise removal and consistency. The data are shaped as 1-s epochs $\times$ channels $\times$ samples for efficiency [33].

The samples for stroke patients are downsampled from 1200 Hz to 200 Hz to reduce computational complexity as usual human neural activities and the damping effects of the skull are at frequencies less than 200 Hz [57, 58]. A $5{th}$ order Butterworth bandpass filter removes non-motor-related frequency components of the signal outside 1 to 40 Hz as this range includes all relevant frequencies for motor processes [59–61]. The flatness response of the Butterworth filter is suitable for preserving the desired frequency range and eliminating irrelevant parts of the signal such as power-line at 50 or 60 Hz [60, 62].

#### Wavelet filter

The automatic tunable artifact removal (ATAR) algorithm is designed to remove artefacts of a signal that does not rely on expert knowledge or manual identification of noisy EEG components like ICA [63]. The ATAR algorithm provides a relatively objective way to remove artefacts from the EEG data by utilising the EEG signal’s intrinsic parameters [63, 64]. The EEG signal is first deconstructed into sets of wavelet coefficients by applying a discrete wavelet transform (DWT) using the Daubechies wavelet 4 (db4) [61, 63, 64]. The decomposition level is 3 for stroke patients to produce coefficients that are approximately associated with the frequency range: 1 to 35 Hz. db4 is relatively smooth which is effective for detecting EEG variations [64]. A linear attenuation filter is applied to either remove or adjust wavelet coefficients that are large according to the following threshold functions [63, 64].

1 $$\begin{aligned} \psi _A\left( r,k_1,k_2\right) = {\left\{ \begin{array}{ll} f_B(r), &{} \text {if } f_B(r) \ge k_1 \\ k_1, &{} \text {otherwise} \end{array}\right. } \end{aligned}$$

and $$\psi _B = 2\psi _A$$,

2 $$\begin{aligned} f_B(r) = k_2e^{B\left( \frac{100r}{2k_2}\right) }, \end{aligned}$$

where *r* is the interquartile range of $\omega$. *B*=0.1 is the attenuation constant (steepness) between 0 and 1. A higher value of $\beta$ makes the ATAR algorithm more aggressive in artefact removal. $\beta$=0.1, the default setting, is close to 0 which prevents loss of signal. $k_1=8$ Hz and $k_2=35$ Hz are the lower and upper frequency bounds to narrow the motor-related components, respectively [61, 63, 64]. The interquartile range of wavelet coefficients, *r*, applied in the threshold function effectively reduce the outliers outside of *r* and retain the core features of the signal [61, 64]. The linear attenuation filter function is given by

3 $$\begin{aligned} \lambda _a\left( \omega ,r,k_1,k_2\right) = {\left\{ \begin{array}{ll} \omega , &{} |\omega |\le \psi _A \\ {{\,\textrm{sgn}\,}}(\omega )\psi _A\left( 1-\frac{|\omega |-\psi _A}{\psi _B-\psi _A}\right) , &{} \psi _A< |\omega |\le \psi _B \\ 0, &{} \text {otherwise}, \\ \end{array}\right. } \end{aligned}$$

where ${{\,\textrm{sgn}\,}}(\cdot )$ is the signum function. Finally, the filtered wavelet coefficients are used to reconstruct the signal by using the inverse wavelet transform.

The attenuation filter function, $\lambda _\alpha$, involves the lower and upper bounds on the threshold value: k1 and k2, respectively[63, 64]. $\beta$ is the attenuation constant (steepness). k1=8 Hz and k2=35 Hz. *r* is the interquartile range of wavelet coefficients[61, 63, 64]. Finally, the first set of the wavelet coefficients is chosen to reconstruct the signal as it is best corresponded with the frequency range: 1 to 40 Hz [61, 63, 64].

### Data analysis

Periodograms of the stroke patients are, respectively, averaged over all epochs for each of the different experimental tasks and are normalised by the minimum–maximum feature scaling function

4 $$\begin{aligned} x'=2\frac{x-x_{min}}{x_{max}-x_{min}}-1 , \end{aligned}$$

with respect to the tasks shown in Fig. 6: VOICE, MI after VOICE, VR+MI, and MI after VR, with 1 = the maximum and – 1 = the minimum. Power spectral densities of the preprocessed signal across frequencies is computed by Welch’s method using a Hamming window with zero padding to smooth the output. Simpson’s rule was used to calculate the band powers in the $\alpha$ and $\beta$ bands by summing the PSD in the respective frequency range [65]. The Wilcoxon signed-rank test was used to compare different pairs of tasks performed by stroke patients with the associated p-values given in Table 1 for the $\alpha$ and $\beta$ bands. The Python MNE library is used to compute the scalp maps for the $\alpha$ and $\beta$ band powers of each stroke patient [66]. The band powers are normalised by Eq. 4, the minimum–maximum feature scaling function, where band powers from all tasks shown in Fig. 6 are considered for each experiment, respectively.

The Python package: antropy is used to compute spectral entropy [67]. Spectral entropy utilises Shannon entropy and the signal’s power spectrum to compute the regularity of the time series corresponding to the uniformity of power spectrum distribution as shown in Eq. 5 [31, 32, 41, 46, 68]:

5 $$\begin{aligned} H_{spec}=-\sum _{f_{0}}^{f_{n}}{\hat{p}}(f)log_{2}({\hat{p}}(f)); {\hat{p}}(f)=\frac{p(f)}{\sum _{f_{0}}^{f_{n}}p(f)}, \end{aligned}$$

where *p*(*f*) is the power spectral density; ${\hat{p}}(f)$ is the normalised power spectral density; $f_{0}$ and $f_{n}$ are, respectively, the first and last frequencies of the integrated frequency range; the logarithmic base is 2 and the spectral entropy is in units of bits. The Python MNE library and Eq. 4 are applied to compute the normalised spectral entropy relative to the experimental tasks specified in Fig. 6 for each stroke patient.

## Supplementary Information

**Additional file 1: Figure S1. **α band power scalp maps of stroke patients 1 to 7 (top row to bottom row) from Experiment 1 showing the intensity variations normalised relative to classes: VOICE, MI after VOICE, MI+VR and MI after VR. Red is 1 = maximum; blue is -1 = minimum. **Figure S2.** β band power scalp maps of stroke patients 1 to 7 (top row to bottom row) from Experiment 1 showing the intensity variations normalised relative to classes: VOICE, MI after VOICE, MI+VR and MI after VR. Red is 1 = maximum; blue is -1 = minimum. **Figure S3.** α band power scalp maps of stroke patients 8 to 16 (top row to bottom row) from Experiment 2 showing the intensity variations normalised relative to classes: VOICE, MI after VOICE and MI after VR. Red is 1 = maximum; blue is -1 = minimum. **Figure S4.** β band power scalp maps of stroke patients 8 to 16 (top row to bottom row) from Experiment 2 showing the intensity variations normalised relative to classes: VOICE, MI after VOICE and MI after VR. Red is 1 = maximum; blue is -1 = minimum. **Figure S5.** Spectral entropy scalp maps of stroke patients 1 to 7 (top row to bottom row) for frequencies 1-40 Hz showing the intensity variations normalised relative to classes from Experiment 1. Red is 1 = maximum; blue is -1 = minimum. **Figure S6.** Spectral entropy scalp maps of stroke patients 8 to 16 (top row to bottom row) for frequencies 1-40 Hz showing the intensity variations normalised relative to classes from Experiment 2, respectively. Red is 1 = maximum; blue is -1 = minimum.

## Data Availability

The dataset of the current study may be requested from the corresponding authors.

## Abbreviations

AO: Action observation
API: Application programming interface
ATAR: Automatic tunable artifacts removal algorithm
BMRS: Brunnstrom motor recovery stages
3D: 3 Dimensional
db4: Daubechies wavelet 4
DWT: Discrete wavelet transform
EEG: Electroencephalogram
ICA: Individual component analysis
MI: Motor imagery
MMSE: Mini-mental state examination
PSD: Power spectral density
VI: Visual imagery
VR: Virtual reality
