# Supplementary material for: Virtual reality and motor imagery for early post-stroke rehabilitation
Source: Biomed Eng Online. 2023 Jul 5;22:66. doi: 10.1186/s12938-023-01124-9 (PMC10320905; doi:10.1186/s12938-023-01124-9)
Supplement: Supplementary file 1 — Additional file 1: Figure S1. α band power scalp maps of stroke patients 1 to 7 (top row to bottom row) from Experiment 1 showing the intensity variations normalised relative to classes: VOICE, MI after VOICE, MI+VR and MI after VR. Red is 1 = maximum; blue is -1 = minimum. Figure S2. β band power scalp maps of stroke patients 1 to 7 (top row to bottom row) from Experiment 1 showing the intensity variations normalised relative to classes: VOICE, MI after VOICE, MI+VR and MI after VR. Red is 1 = maximum; blue is -1 = minimum. Figure S3. α band power scalp maps of stroke patients 8 to 16 (top row to bottom row) from Experiment 2 showing the intensity variations normalised relative to classes: VOICE, MI after VOICE and MI after VR. Red is 1 = maximum; blue is -1 = minimum. Figure S4. β band power scalp maps of stroke patients 8 to 16 (top row to bottom row) from Experiment 2 showing the intensity variations normalised relative to classes: VOICE, MI after VOICE and MI after VR. Red is 1 = maximum; blue is -1 = minimum. Figure S5. Spectral entropy scalp maps of stroke patients 1 to 7 (top row to bottom row) for frequencies 1-40 Hz showing the intensity variations normalised relative to classes from Experiment 1. Red is 1 = maximum; blue is -1 = minimum. Figure S6. Spectral entropy scalp maps of stroke patients 8 to 16 (top row to bottom row) for frequencies 1-40 Hz showing the intensity variations normalised relative to classes from Experiment 2, respectively. Red is 1 = maximum; blue is -1 = minimum. [file 12938_2023_1124_MOESM1_ESM.pdf]

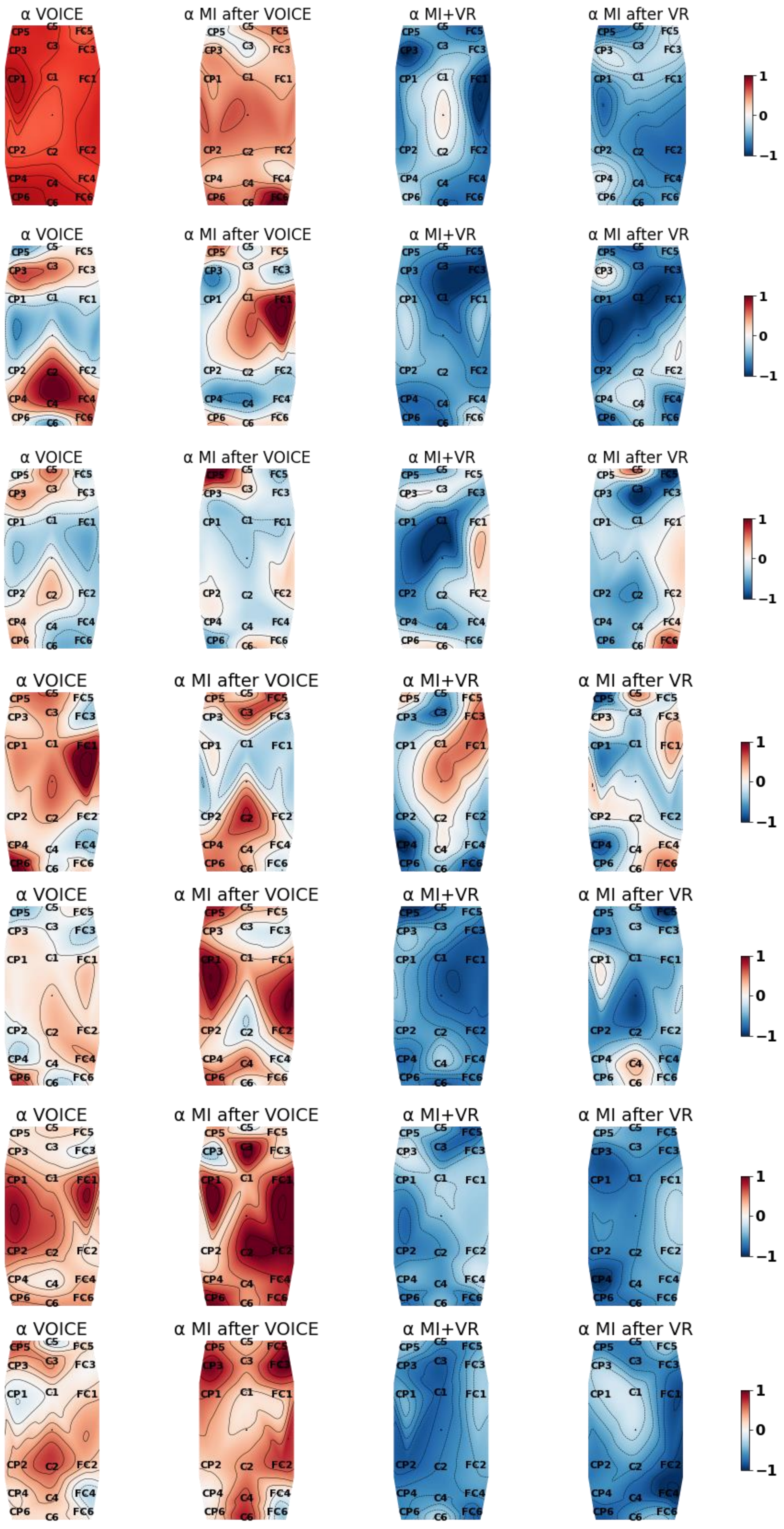

Figure 1: α band power scalp maps of stroke patients 1 to 7 (top row to bottom row) from Experiment 1 showing the intensity variations normalised relative to classes: VOICE, MI after VOICE, MI+VR and MI after VR. Red is 1 = maximum; blue is -1 = minimum.

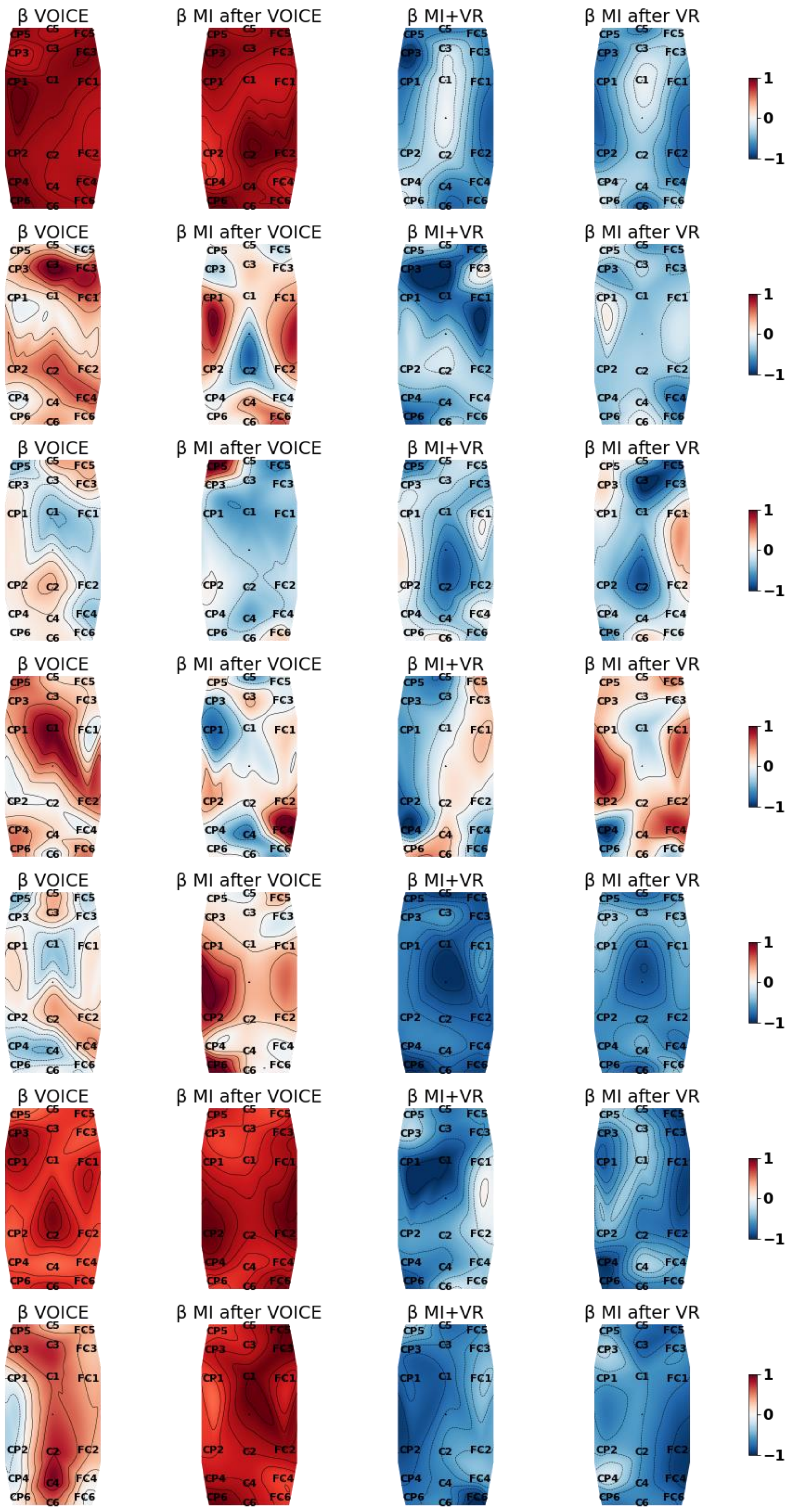

Figure 2: β band power scalp maps of stroke patients 1 to 7 (top row to bottom row) from Experiment 1 showing the intensity variations normalised relative to classes: VOICE, MI after VOICE, MI+VR and MI after VR. Red is 1 = maximum; blue is -1 = minimum.

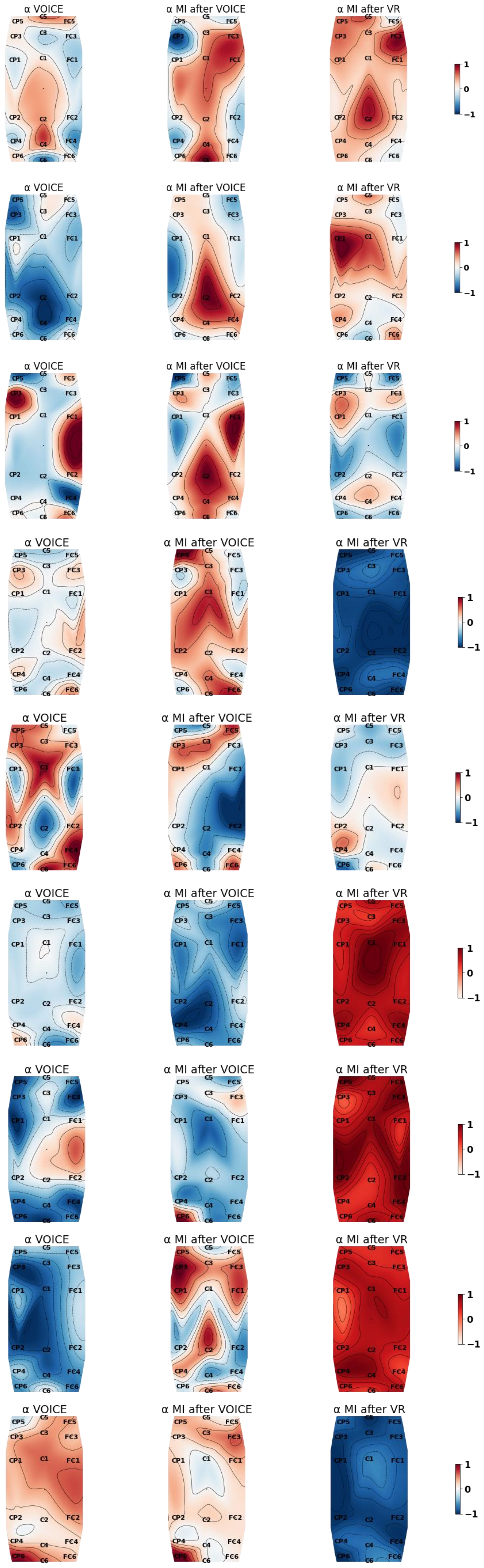

Figure 3: α band power scalp maps of stroke patients 8 to 16 (top row to bottom row) from Experiment 2 showing the intensity variations normalised relative to classes: VOICE, MI after VOICE and MI after VR. Red is 1 = maximum; blue is -1 = minimum.

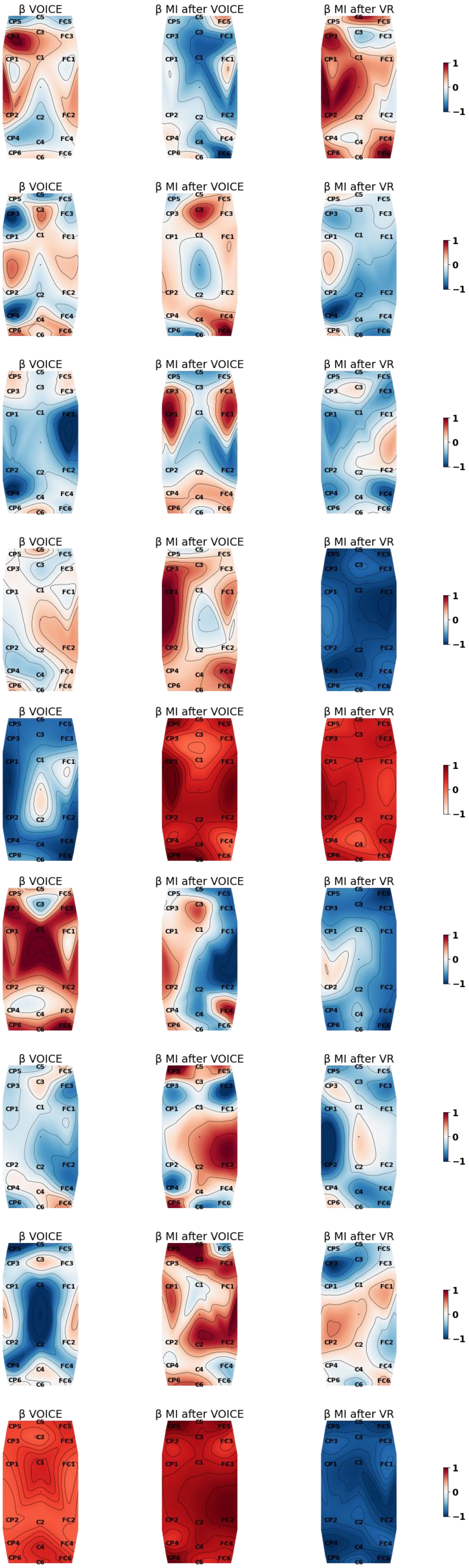

Figure 4: β band power scalp maps of stroke patients 8 to 16 (top row to bottom row) from Experiment 2 showing the intensity variations normalised relative to classes: VOICE, MI after VOICE and MI after VR. Red is 1 = maximum; blue is -1 = minimum.

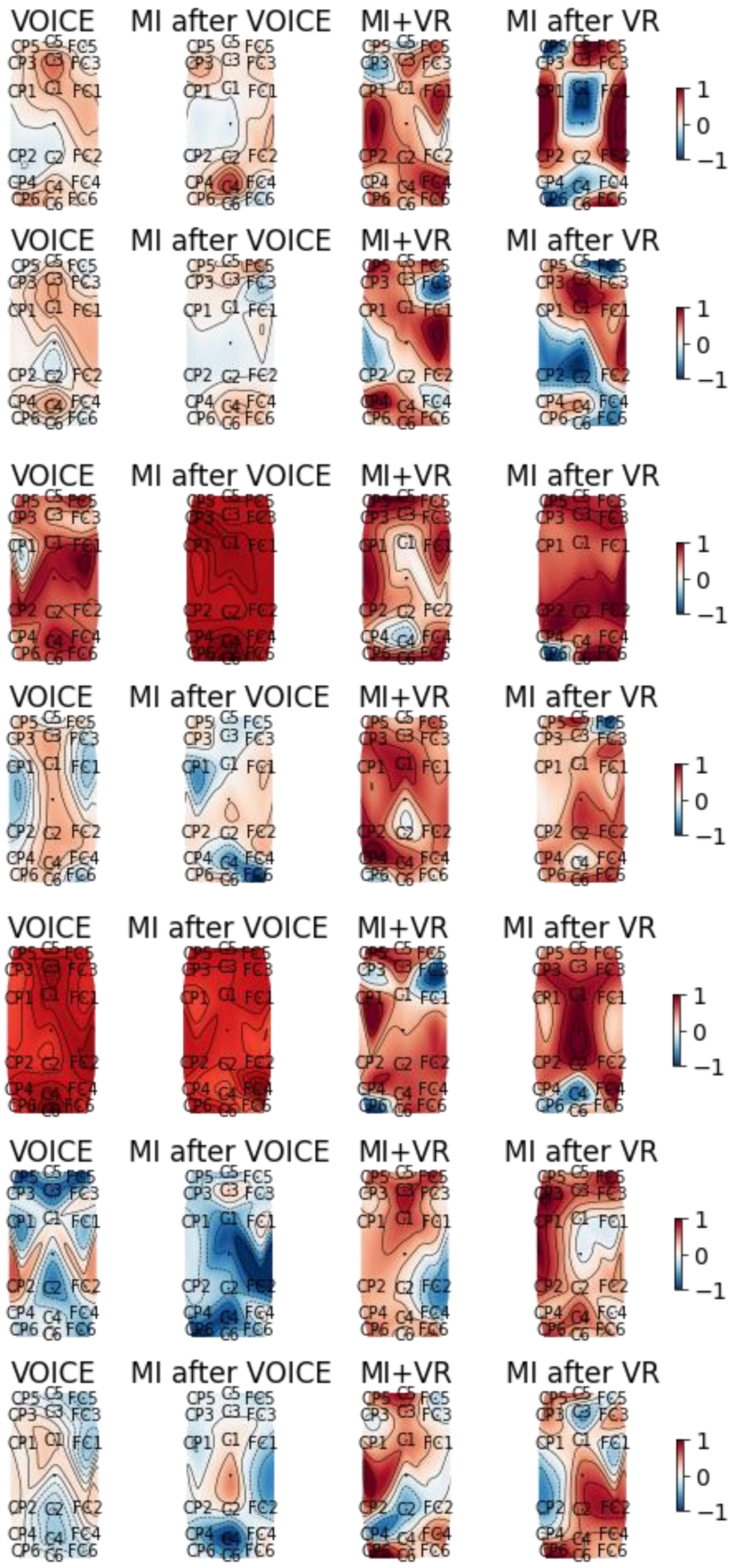

Figure 5: Spectral entropy scalp maps of stroke patients 1 to 7 (top row to bottom row) for frequencies 1-40 Hz showing the intensity variations normalised relative to classes from Experiment 1. Red is 1 = maximum; blue is -1 = minimum.

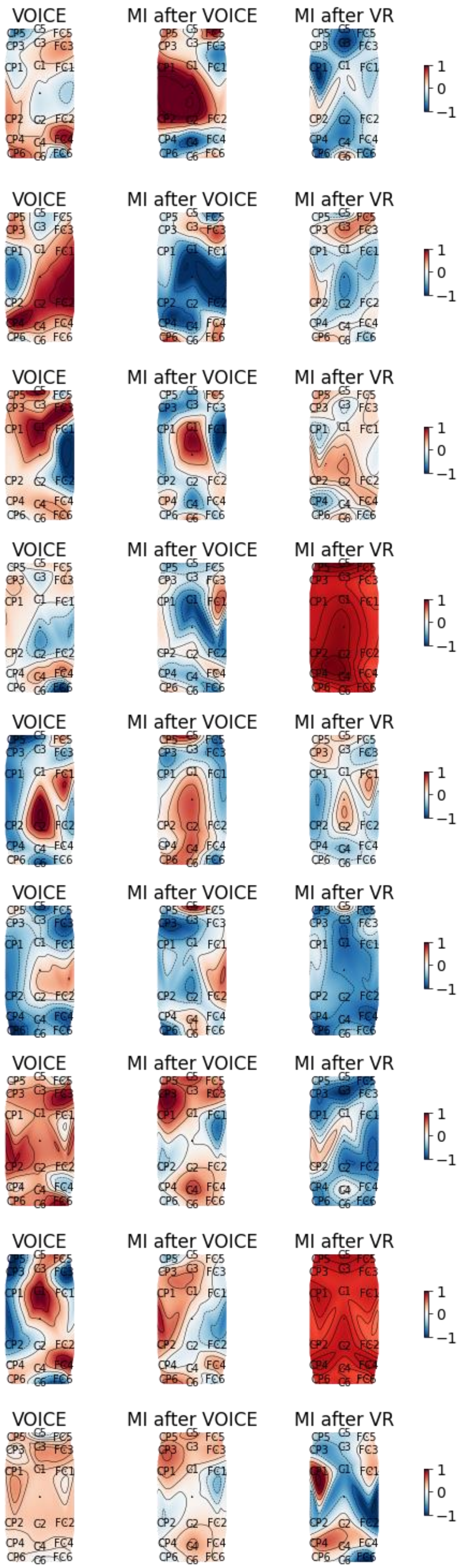

Figure 6: Spectral entropy scalp maps of stroke patients 8 to 16 (top row to bottom row) for frequencies 1-40 Hz showing the intensity variations normalised relative to classes from Experiment 2, respectively. Red is 1 = maximum; blue is -1 = minimum.
